# A favorable outcome of intensive immunotherapies for new-onset refractory status epilepticus (NORSE)

**DOI:** 10.1186/s40560-018-0315-7

**Published:** 2018-07-31

**Authors:** Satoshi Kodama, Noritoshi Arai, Akiyoshi Hagiwara, Akio Kimura, Sousuke Takeuchi

**Affiliations:** 1 Department of Neurology, Center Hospital of the National Center for Global Health and Medicine, 1-21-1 Toyama, Shinjuku-ku, Tokyo, 162-8655 Japan; 2 Department of Emergency Medicine and Critical Care, Center Hospital of the National Center for Global Health and Medicine, 1-21-1 Toyama, Shinjuku-ku, Tokyo, 162-8655 Japan

**Keywords:** Status epilepticus, New-onset refractory status epilepticus (NORSE), Immunotherapy, Autoimmune encephalitis, Plasma exchange (PE), Intravenous immunoglobulin (IVIG)

## Abstract

**Background:**

New-onset refractory status epilepticus (NORSE) is a newly defined critical disease entity characterized by prolonged periods of refractory epileptic seizure with no readily identifiable cause in otherwise healthy individuals. Its etiology is uncertain, but autoimmune encephalitis is a possible candidate for the underlying cause of this condition. Immunotherapies could be considered for this condition, but its efficacy is not established.

**Case presentation:**

A 31-year-old man with no prior history presented with refractory status epilepticus. His seizure persisted even with multiple anti-epileptic drugs and required prolonged general anesthesia under mechanical ventilation. Magnetic resonance imaging and cerebrospinal fluid did not indicate the cause of seizure, and autoantibodies related to encephalitis were not detected. It was speculated that the patient had occult autoimmune encephalopathy because of its acute-onset clinical course preceded by fever, even without definite evidence of an autoimmune mechanism. The patient received intravenous methylprednisolone, plasma exchange, and intravenous immunoglobulin in succession and manifested a favorable outcome after these treatments.

**Conclusion:**

Our case supports the efficacy of immunotherapies for NORSE even though it does not manifest definite evidence for autoimmune background. Clinicians should consider these immunotherapies for NORSE as early as possible, because this condition is associated with high mortality and morbidity owing to prolonged seizure activity and long-term intensive care including general anesthesia and mechanical ventilation.

## Background

Status epilepticus (SE) is an important neurological condition for emergency physicians and intensivists because it could damage brain function irreversibly unless treated promptly and effectively. Generally, anti-epileptic drugs (AEDs) are used for this condition at first, but it sometimes show resistance to AEDs and requires long-term intensive care, in several weeks to months, including general anesthesia and mechanical ventilation until seizure activities subside. New-onset refractory status epilepticus (NORSE) is characterized by prolonged periods of refractory epileptic seizure with no readily identifiable cause in otherwise healthy individuals [1]. NORSE is a rare but critical neurological condition because it shows considerable high mortality and morbidity. Recently, some case series have suggested the majority of its etiology is autoimmune encephalitis and immunotherapies could be a choice even without evidence of autoantibodies. However, the efficacy of immunotherapies for NORSE is still controversial.

We experienced a case of NORSE which was treated effectively with intensive immunotherapy including intravenous methylprednisolone (IVMP), plasma exchange (PE), and intravenous immunoglobulin (IVIG). Our case could provide a profound insight into pathophysiology and management of this condition.

## Case presentation

**Fig. 1:**
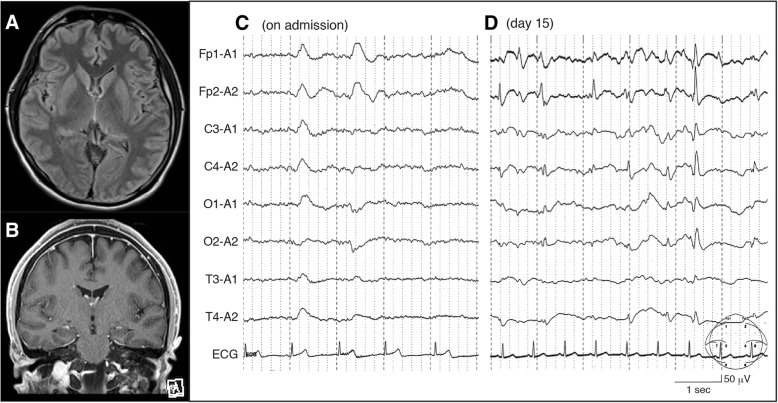
**a**, **b** Brain magnetic resonance imaging (MRI) on admission did not show intracranial abnormality on fluid attenuated inverse recovery (FLAIR) (**a**) or gadolinium enhancement (**b**). **c**, **d** Interictal electroencephalography on admission demonstrated bilateral intermittent delta activity predominantly in bilateral frontal region (**c**), and periodic epileptiform discharges predominantly in the bilateral frontal area were seen every 0.5–1.0 s on day 15, suggesting continuing seizure activity

A 31-year-old man with no medical history was presented to our emergency department (ED) with disturbance of consciousness and generalized seizure. After having a fever, he had been out of contact for 3 days and his colleague found him unresponsive in his apartment. Soon after arriving at the ED, he showed generalized tonic-clonic seizure (GTCS) starting from his left limbs, which ceased after intravenous diazepam 10 mg. Weakness, pyramidal signs, and meningeal irritation signs were not seen. Laboratory examination showed systemic inflammation: white blood cells 26,100/μL and C-reactive protein 8.56 mg/dL. Creatinine was 1.69 mg/dL, urea nitrogen was 41.0 mg/dL, and creatine kinase was 60,264 IU/mL, showing dehydration and rhabdomyolysis presumably due to prolonged impaired consciousness. Lumber puncture was unremarkable except for increased opening pressure (30 cmH_2_O): cells 2.4/μL, protein 26 mg/dL, glucose 97 mg/dL, and IgG 2.0 mg/dL. Culture of cerebrospinal fluid was negative. Serum HIV, herpes simplex virus, and varicella zoster virus antibodies were negative. Anti-nuclear, anti-double-stranded DNA, anti-glutamic acid decarboxylase, anti-thyroid peroxidase, anti-thyroglobulin, and anti-neutrophilic cytoplasmic antibodies were negative as well. Magnetic resonance imaging (MRI) showed no intracranial lesion or abnormal gadolinium enhancement (Fig. 1a, b). Interictal electroencephalogram showed generalized periodic delta waves predominantly on the bilateral frontal areas (Fig. 1c).

Despite administering 1000 mg of fosphenytoin for the seizure, he repeated GTCS on day 2. He was intubated and mechanically ventilated on that day due to GTCS accompanied with respiratory depression. Although valproate 900 mg through the nasogastric tube and intravenous propofol was started and the dose of propofol was gradually increased, GTCS recurred on day 5 and levetiracetam 1000 mg and intravenous midazolam was added to control the seizures. Even with these medications he repeated GTCS during day 7 through 14, which obliged us to increase the dose of midazolam to the maximum and levetiracetam to 3000 mg, and also add carbamazepine 400 mg and zonisamide 600 mg. Tracheotomy was performed on day 15 because of prolonged mechanical ventilation. During days 15–19, EEG showed periodic sharp discharges predominantly on the bilateral frontal regions every 0.5–1.0 s even during the patient did not manifest apparent seizure (Fig. 1d), suggesting the condition of non-convulsive status epilepticus (NCSE). Propofol was transferred to thiopental and its dose was increased to the level that leads to a burst and suppression pattern on EEG during days 20–25.

**Fig. 2:**
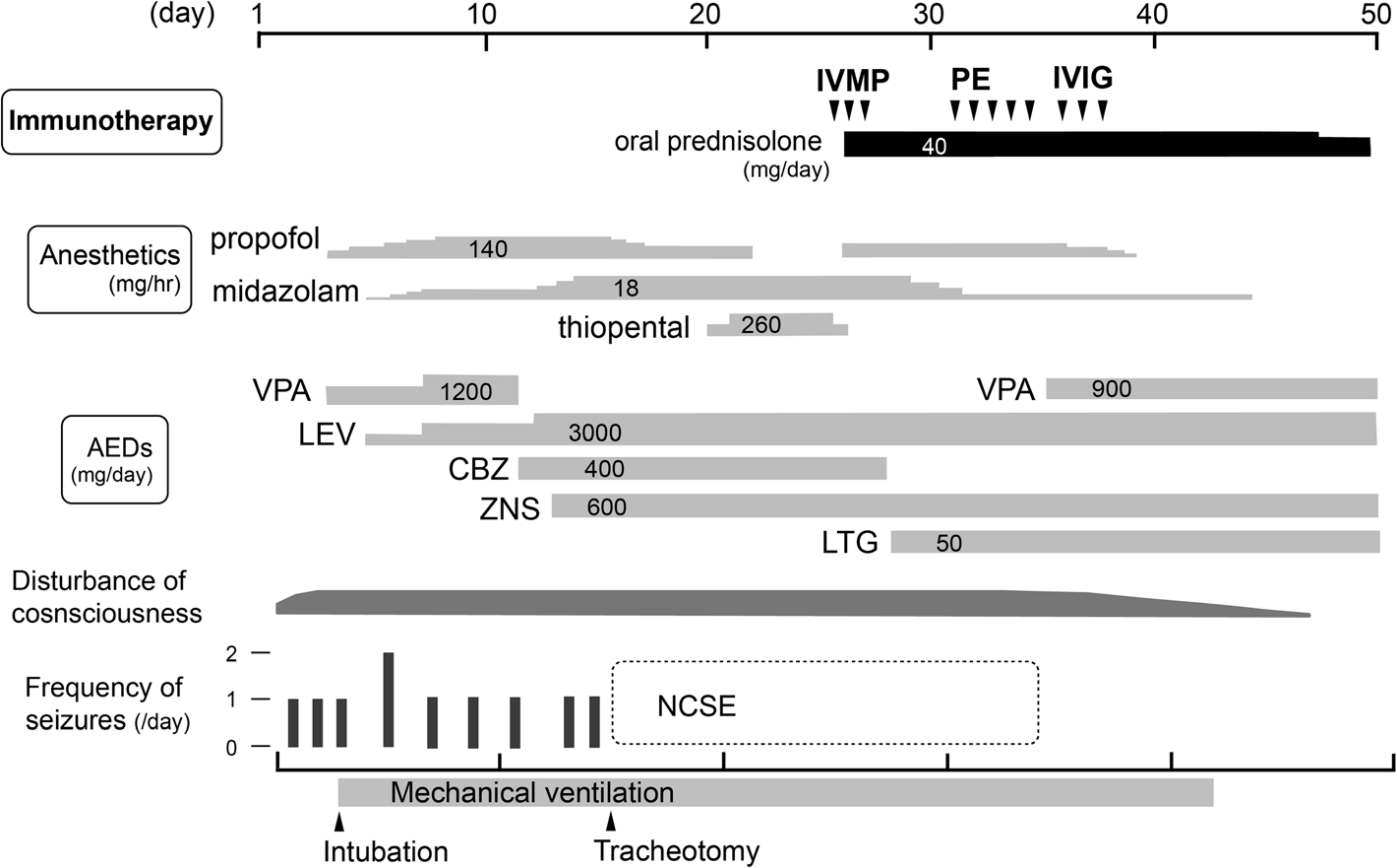
After administration of plasma exchange, the patient gradually regained consciousness and eventually discontinued long-term mechanical ventilation and general anesthesia. IVMP, intravenous methylprednisolone; PE, plasma exchange; IVIG, intravenous immunoglobulin; VPA, sodium valproate; LEV, levetiracetam; CBZ, carbamazepine; ZNS, zonisamide; LTG, lamotrigine

During this period, repeat head MRI and lumber puncture showed no cause of the status epilepticus. Chest and abdominal computed tomography (CT) and positron emission tomography (PET) did not represent neoplastic lesions. Based on several recent reports, we presumed some occult autoimmune disorders were behind this patient’s refractory status epilepticus and immunomodulative therapy could be effective. The patient received intravenous methylprednisolone (IVMP) on days 25–27 (methylprednisolone 1000 mg/day for 3 days), but his EEG continued to manifest epileptic discharge when the general anesthesia was tapered. We administered plasma exchange (PE) on days 31–35 and intravenous immunoglobulin therapy (IVIG) on days 36–38 (0.4 g/kg for 3 days), under the informed consent of his family (Fig. 2). He came to show gradual improvement of consciousness and decreased epileptic discharge on EEG around day 35, making it possible to taper the anesthesia. Mechanical ventilation was discontinued on day 42, and the sedative agents for general anesthesia were withdrawn by day 44.

The patient gradually regained his ability of daily life and showed well-preserved memory function and his computer skills for the job, but mild anterograde amnesia, irritability, and difficulty in concentration remained. Although the patient was discharged home on day 189, refractory focal onset seizures with impaired awareness were seen every 2–3 days which required valproate 1200 mg, levetiracetam 3000 mg, zonisamide 400 mg, lamotrigine 200 mg, and clobazam 7.5 mg.

## Discussion

Our patient showed new-onset refractory epileptic seizure subsequent to a febrile episode, which was reluctant to resolve with combination of multiple AEDs and general sedative agents. The clinical course was compatible with NORSE considering prolonged status epilepticus without no prior history of epilepsy and absence of identifiable causative factors. Although NORSE was initially defined as not a proven etiology of epilepsy [1], some recent studies have suggested autoimmune encephalitis may be a common cause of this condition [2, 3]. Based on this estimation, immunotherapies are administered in an increasing number of NORSE cases even without detection of specific antibodies regarding autoimmune encephalitis [3].

However, there is a dilemma in initiating immunotherapies in NORSE. Although our case showed favorable outcome, the effectiveness of immunotherapy for NORSE is not established due to lack of evidence, rare prevalence, and ethical obstacle in performing randomized controlled trials. Early case studies showed no response to immunotherapy [1], and only a small number of recent case studies have suggested the occasional effectiveness of IVMP, IVIG, and PE for cryptogenic NORSE [2, 4–7]. The clinicians hesitate to perform these potentially hazardous immune-suppressing treatments in the absence of well-established inflammatory causes [3]. On the other hand, it is reported that mortality of NORSE is up to 22%, and 39% of patients show a poor neurologic outcome [3]. Considering its high mortality and morbidity, the use of immunotherapies should be considered in NORSE even without concrete evidence of an autoimmune etiology when seizure is not controlled with conventional anti-epileptic drugs and sedative agents.

It is also difficult when to administer immunotherapies in NORSE. In our case, we administered IVMP as the first immunotherapy after 25 days from the onset of seizure. We initially refrained from immunotherapy because autoimmune etiology seemed less likely with negative MRI and CSF findings and there was also a concern that it might aggravate the patient’s condition if he had an infectious etiology. However, the prolonged seizure activity even under intensive anti-epileptic agents eventually pushed us to administering the immunotherapy. As a result, the patient fully recovered functionally but sustained refractory focal seizure requiring multiple anti-epileptic drugs. In general, prolonged seizure activity can aggravate the epileptogenecity by a kindling mechanism [8]. In that point, earlier immunotherapies could have prevented the patient’s late seizures. Several studies on autoimmune encephalitis showed early treatment is associated with better outcomes [9, 10]. Besides, prolonged mechanical ventilation and general anesthesia which are often required for persisting seizures could expose the patients to the risk of systemic complications and increased mortality due to pneumonia, hepatic injury, and cardiac suppression. Considering these aspects, immunotherapies should not be postponed when clinicians diagnose NORSE.

Yet, there is no clear consensus on which cases are the good candidates for these treatments. Dubey et al. proposed Antibody Prevalence in Epilepsy (APE) score and Response to Immunotherapy in Epilepsy (RITE) score, based on the clinical characteristics and the neural antibody evaluation of epilepsy patients in Mayo Clinic [11]. In that study, the scores calculated with several clinical features such as new-onset epilepsy, autonomic dysfunction, viral prodrome, faciobrachial dystonic seizures/oral dyskinesia, inflammatory CSF profile, and mesial temporal MRI abnormalities had a significant association with positive antibody results. High ratings of these scores are associated with high possibilities of autoimmune etiology and efficacy of immunotherapies. These scores could help clinicians consider the validity of immunotherapies for NORSE.

Although IVMP, IVIG, and PE are the most frequently used immunotherapies for NORSE, it is still uncertain which modality is the most effective because no clinical trial had been performed and each treatment had been administered with different doses and durations in the past case studies, making it difficult to compare each outcome. In practice, these immunotherapies are often tried sequentially when one of them fail to improve the patient’s condition. In our case, we chose IVMP as the first choice because of its relatively low side effect profile and easy availability, and considered PE and IVIG as a second- or third-line. It is even more difficult to tell which would be more effective, PE or IVIG, because both of them show equivalent therapeutic effects on several other neuroimmunological disorders such as Guillain-Barre syndrome [12] and myasthenia gravis [13]. We administered PE prior to IVIG, because infused immunoglobulin of IVIG therapy could be eliminated by PE if these treatments were performed in the opposite order. However, it should be noted PE is not suitable for patients with infection or hemodynamically unstable status because it uses extracorporeal circulation. NORSE patients are often in critically ill status because of prolonged anesthesia and ventilation; therefore, clinicians should evaluate which treatment would be appropriate for each clinical condition. Rituximab [2], cyclophosphamide [2, 14], ketamine [2, 15], and vagal nerve stimulation [16] have been reported as other possible treatment options for NORSE so far and should be considered if initial treatments are not effective.

Although it became more common to start immunotherapies without a proven etiology, it remains still important to investigate the cause of NORSE because it also can justify the use of intensive immunotherapies and help consider further treatment. Gaspart et al. reported that additional investigation revealed that autoimmune encephalitis with specific autoantibodies accounted for 48 out of 130 (37%) initially cryptogenic NORSE cases [3]. We excluded anti-*N*-methyl-D-aspartate (NMDA) antibody in our case, but other antibodies that could have triggered encephalitis remained unmeasured (e.g., antibodies against leucine-rich, glioma-inactivated 1 (LGI1), contactin-associated protein-like 2 (CASPR2)). These antibodies bind to extracellular epitopes on the cell surface and are often associated with good responsibility for the treatments. Considering its preferable outcome and negative MRI and CSF findings, it is presumable that our case was associated with antibodies against cell-surface antigens, rather than those against intracellular proteins in which neuronal loss is more frequent [17]. It should be noted, however, that several antibodies against cell-surface antigen are often associated with neoplasm (e.g., antibodies against gamma-aminobutyric acid type B receptor (GABAB), alpha-amino-3-hydroxy-5-methyl-4isoxazolepropionic acid receptor (AMPA)) and tend to show poorer outcome than other cell-surface antibodies [17]. Thorough exploration of the neoplasm is also important in NORSE. There was also a possibility that unknown autoantibodies were related to our case, because our clinical characteristics were not necessarily corresponding to encephalitis with those known antibodies mentioned above. Immunostaining of rat brain using patient’s serum was also reported to be effective to prove autoimmune pathogenesis with unknown etiology [18]. Such extensive investigations should be considered in NORSE.

## Conclusion

We described a case of NORSE which showed favorable outcome with intensive immunotherapy. Intensivists should be familiar with this disease entity, because it could require long-term general anesthesia and mechanical ventilation while it could be reversible with immunotherapy.

## Abbreviations

AEDs: Antiepileptic drugs
CT: Computed tomography
ED: Emergency department
GTCS: Generalized tonic-clonic seizure
IVIG: Intravenous immunoglobulin
IVMP: Intravenous methylprednisolone
MRI: Magnetic resonance imaging
NORSE: New-onset refractory status epilepticus
PE: Plasma exchange
PET: Positron emission tomography
SE: Status epilepticus
